# UV RESISTANCE LOCUS 8–Mediated UV-B Response Is Required Alongside CRYPTOCHROME 1 for Plant Survival in Sunlight under Field Conditions

**DOI:** 10.1093/pcp/pcad113

**Published:** 2023-09-27

**Authors:** Reinhold Stockenhuber, Reiko Akiyama, Nicolas Tissot, Stefan Milosavljevic, Misako Yamazaki, Michele Wyler, Adriana B Arongaus, Roman Podolec, Yasuhiro Sato, Alex Widmer, Roman Ulm, Kentaro K Shimizu

**Affiliations:** Department of Evolutionary Biology and Environmental Studies, University of Zurich, Winterthurerstrasse 190, Zurich 8057, Switzerland; Department of Evolutionary Biology and Environmental Studies, University of Zurich, Winterthurerstrasse 190, Zurich 8057, Switzerland; Department of Plant Sciences, Section of Biology, Faculty of Sciences, University of Geneva, 30 Quai E. Ansermet, Geneva 1211, Switzerland; Institute of Genetics and Genomics of Geneva (iGE3), University of Geneva, Geneva 1211, Switzerland; Department of Evolutionary Biology and Environmental Studies, University of Zurich, Winterthurerstrasse 190, Zurich 8057, Switzerland; SIB Swiss Institute of Bioinformatics, University of Zurich, Winterthurerstrasse 190, Zurich 8057, Switzerland; Department of Evolutionary Biology and Environmental Studies, University of Zurich, Winterthurerstrasse 190, Zurich 8057, Switzerland; Department of Evolutionary Biology and Environmental Studies, University of Zurich, Winterthurerstrasse 190, Zurich 8057, Switzerland; Department of Plant and Microbial Biology, University of Zurich, Zollikerstrasse 107, Zurich 8008, Switzerland; Department of Plant Sciences, Section of Biology, Faculty of Sciences, University of Geneva, 30 Quai E. Ansermet, Geneva 1211, Switzerland; Institute of Genetics and Genomics of Geneva (iGE3), University of Geneva, Geneva 1211, Switzerland; Department of Plant Sciences, Section of Biology, Faculty of Sciences, University of Geneva, 30 Quai E. Ansermet, Geneva 1211, Switzerland; Institute of Genetics and Genomics of Geneva (iGE3), University of Geneva, Geneva 1211, Switzerland; Department of Evolutionary Biology and Environmental Studies, University of Zurich, Winterthurerstrasse 190, Zurich 8057, Switzerland; Institute of Integrative Biology, ETH Zurich, Universitätstrasse 16, Zurich 8092, Switzerland; Department of Plant Sciences, Section of Biology, Faculty of Sciences, University of Geneva, 30 Quai E. Ansermet, Geneva 1211, Switzerland; Institute of Genetics and Genomics of Geneva (iGE3), University of Geneva, Geneva 1211, Switzerland; Department of Evolutionary Biology and Environmental Studies, University of Zurich, Winterthurerstrasse 190, Zurich 8057, Switzerland; Kihara Institute for Biological Research, Yokohama City University, 641-12 Maioka, Totsuka-ward, Yokohama 244-0813, Japan

**Keywords:** Abiotic stress, *Arabidopsis thaliana*, Common garden, Photoreceptor, Survival, UV attenuation

## Abstract

As sessile, photoautotrophic organisms, plants are subjected to fluctuating sunlight that includes potentially detrimental ultraviolet-B (UV-B) radiation. Experiments under controlled conditions have shown that the UV-B photoreceptor UV RESISTANCE LOCUS 8 (UVR8) controls acclimation and tolerance to UV-B in *Arabidopsis thaliana*; however, its long-term impact on plant fitness under naturally fluctuating environments remain poorly understood. Here, we quantified the survival and reproduction of different *Arabidopsis* mutant genotypes under diverse field and laboratory conditions. We found that *uvr8* mutants produced more fruits than wild type when grown in growth chambers under artificial low-UV-B conditions but not under natural field conditions, indicating a fitness cost in the absence of UV-B stress. Importantly, independent double mutants of *UVR8* and the blue light photoreceptor gene *CRYPTOCHROME 1* (*CRY1*) in two genetic backgrounds showed a drastic reduction in fitness in the field. Experiments with UV-B attenuation in the field and with supplemental UV-B in growth chambers demonstrated that UV-B caused the *cry1 uvr8* conditional lethal phenotype. Using RNA-seq data of field-grown single and double mutants, we explicitly identified genes showing significant statistical interaction of *UVR8* and *CRY1* mutations in the presence of UV-B in the field. They were enriched in Gene Ontology categories related to oxidative stress, photoprotection and DNA damage repair in addition to UV-B response. Our study demonstrates the functional importance of the UVR8-mediated response across life stages *in natura*, which is partially redundant with that of cry1. Moreover, these data provide an integral picture of gene expression associated with plant responses under field conditions.

## Introduction

Plants must cope with changing environments to survive and reproduce. Field studies have shown that the functions of a few or even single genes affect fitness components, namely, biomass and fruit production (Külheim et al. 2002, Tian et al. 2003, Kerwin et al. 2017, Taylor et al. 2019). Recently, more studies have shown that plant gene expression patterns and phenotypes observed in the laboratory are often different from those in natural environments (Shimizu et al. 2011, Kudoh 2016, Kerwin et al. 2017, Yamasaki et al. 2017, Song et al. 2018, Sato et al. 2019b). One environmental factor for which such differences apply is light. Indeed, photoreceptor-mediated perception and responses to solar radiation contribute to plant survival and reproduction in the field (Yanovsky et al. 1995, Galen et al. 2004, Liu et al. 2004, Mazza and Ballaré 2015, Moriconi et al. 2018, Rai et al. 2019, Sellaro et al. 2019) and thereby provide a key to understanding plant adaptation to naturally fluctuating environments.

*Arabidopsis thaliana* (abbreviated as Arabidopsis hereafter) has 13 photoreceptors from five distinct gene families that sense the light environment, namely, five red/far-red light–perceiving phytochromes (phyA–E); seven blue/UV-A photoreceptors, comprising two cryptochromes (cry1 and cry2), three Zeitlupe family members (ztl, fkf1 and lkp2), two phototropins (phot1 and phot2) and the UV-B photoreceptor UV RESISTANCE LOCUS 8 (UVR8) (Rizzini et al. 2011, Galvão and Fankhauser 2015, Podolec et al. 2021a). UV-B is a potentially damaging abiotic stress factor that may affect survival and thus the fitness and distribution of plant populations (Escobar-Bravo et al. 2017, Jenkins 2017, Demarsy et al. 2018). UVR8 orchestrates UV-B-induced photomorphogenesis and stress acclimation in plants (Podolec et al. 2021a). The fundamental role of UVR8 was shown in both controlled chamber conditions and sun simulators that mimic natural sunlight, in which pronounced adverse effects were observed for the phenotypes of *uvr8* mutants and their survival (Kliebenstein et al. 2002, Brown et al. 2005, Favory et al. 2009). By contrast, in common gardens in Finland and Ireland, Arabidopsis plants defective in *UVR8* did not show excess mortality at the seedling stage or an obvious aberrant morphology although they did display reduced photoprotective pigment levels (Morales et al. 2013, Coffey et al. 2017). Similarly, *UVR8* RNAi mutants of *Nicotiana attenuata* grown in the Great Basin Desert (Utah, USA) showed only mild phenotypic changes in photosynthetic rate and microbial community composition (Santhanam et al. 2017). Despite recent progress in understanding the UVR8 molecular mechanism (Podolec et al. 2021a), the effects of the *UVR8* gene on plant fitness remain poorly understood under field conditions.

Recent studies demonstrate overlapping signaling mechanisms and partially redundant functions of UVR8 with other photoreceptors, especially with cry1 (Podolec and Ulm 2018, Lau et al. 2019, Ponnu et al. 2019, Rai et al. 2019, 2020, Tissot and Ulm 2020, Wang and Lin 2020). Some of these studies showed defective growth of a *cry1 cry2 uvr8* triple mutant under sunlight and presented an RNA-seq analysis of the *uvr8-2* mutant alongside that of the *cry1 cry2* mutant after a short exposure to sunlight (Rai et al. 2019, 2020). However, little is known about the long-term impacts of these genes on plant fitness and the genetic interaction of *UVR8* and *CRYPTOCHROME 1* (*CRY1*) on gene expression under long-term growth *in natura*. For an *in natura* understanding of photoreceptors, it is necessary to quantify plant fitness and gene expression under various environmental conditions.

In this study, we investigated plant survival and reproduction of *uvr8* mutants under diverse field and laboratory conditions. We also analyzed how the potential overlap of photoreceptor function between UVR8 and cry1 affects fitness components. Furthermore, we conducted RNA-seq to examine gene expression changes under field and laboratory conditions. By quantitatively assessing the fitness components and underlying molecular mechanisms of different mutants in various environments, we addressed the following questions:

- Are fitness components (i.e. survival and reproductive output) associated with the UVR8-mediated response?
- Do *cry1* and *uvr8* mutations have synergistic effects on fitness in the field?
- Which genes are up- or downregulated in the *cry1 uvr8* double mutant under long exposure to sunlight? Which genes show interaction effects between *cry1* and *uvr8* mutations regarding their expression?

## Results

### UVR8-mediated UV-B response under growth chamber conditions is associated with a fitness reduction

**Fig. 1:**
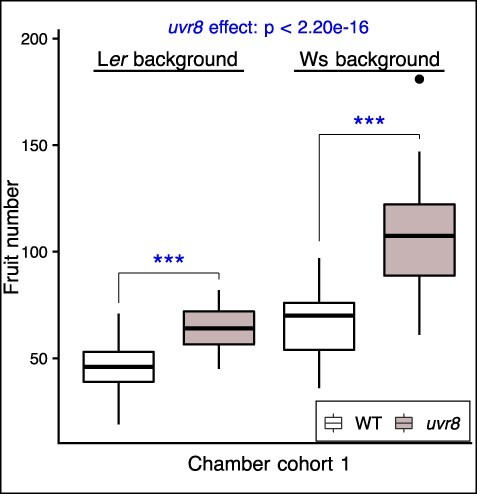
Fitness components of *uvr8* mutants in a growth chamber. Boxplot of fruit number in *uvr8* null mutants in two backgrounds, comparing WT (white) and *uvr8*-mutant (*uvr8*, shaded) plants of two genetic backgrounds under chamber conditions (chamber cohort 1). Genotypes used and number of replicates (left to right, as plotted): WT (L*er*), *n* = 28; *uvr8* (*uvr8-1*), *n* = 28; WT (Ws), *n* = 28; *uvr8* (*uvr8-7*), *n* = 28. Statistically significant differences indicated by asterisks are based on ANOVA of LMs explaining the fruit number by *UVR8* function for each subset of data by background (**Supplementary Table S3**). Bars indicate the median, and boxes indicate the interquartile range. Whiskers extend to the most extreme data point that is not more than 1.5 times the interquartile range from the box, and an outlier is indicated with a dot; *** *P* ≤ 0.001. The *P*-value for the *UVR8* effect over both backgrounds is given at the top of the plot. Further fruit-trait fitness components in chamber environments are presented in **Supplementary Fig. S1**.

To examine how the UVR8-mediated UV-B response affects fitness components, three independent *uvr8* null mutants and their respective wild types (WTs) (*uvr8-1* in the Landsberg *erecta* (L*er*) background, *uvr8-7* in Wassilewskija (Ws) and *uvr8-19* in Col-0; **Supplementary Table S1**) were grown in standard growth chambers and the reproductive output and growth of plants of each genotype were analyzed (**Supplementary Tables S2, S3**). The growth chambers had constant, low levels of UV-B supplied with fluorescent white light tubes (‘Chamber-UV’), providing approximately 1.5% of the daily UV-B present in the field in summer (**Supplementary Table S4**). The *uvr8* mutants produced significantly more fruits (also called siliques) than the WTs (*P* < 2.20 × 10^−16^, **Fig. 1**; *P* < 1.43 × 10^−6^, **Supplementary Fig. S1A**). Because no significant differences in fruit length (*P* = 0.532, **Supplementary Fig. S1B**) or seeds per fruit (*P* = 0.578, **Supplementary Fig. S1C**) were observed, the per-plant seed number was thus increased. Similarly, the *uvr8* mutants produced more overall aboveground biomass (fresh weight), which is an indicator of growth, than the WTs (*P* = 0.014, **Supplementary Fig. S1D**). By contrast, no significant difference in reproductive output was detected for plants grown in the standard growth chamber when UV-B was filtered out (‘Chamber-UV-B exclusion’ conditions; *P* = 0.201, **Supplementary Fig. S1E**). Taken together, these results suggest that a significant reduction in fitness is associated with low levels of UV-B and the corresponding response mediated by functional alleles of *UVR8* under growth chamber conditions.

### Fitness components of single *uvr8* mutants under diverse field conditions

We conducted experiments under field conditions in Zurich, a location representing the natural range of *Arabidopsis thaliana*, to investigate whether *uvr8* mutants show altered fitness in sunlight. Although Arabidopsis is often considered to overwinter (i.e. seeds germinate in fall, plants remain at the rosette stage during winter, and flower in spring, also known as the winter annual habit), non-overwintering plants were reported as well (seeds germinate in spring/summer and flower in summer/fall, also known as the summer annual habit) (Thompson 1994, Donohue 2002, Picó 2012, Pescheck and Bilger 2019). To schedule experiments in Zurich, we determined the Arabidopsis growth season from herbarium specimens and field observations. Among 116 specimens collected in or near the canton of Zurich deposited in Zurich Herbaria, nine had flowers and/or fruits in July and August, indicating non-overwintering (**Supplementary Fig. S2A**), whereas others flowered in early spring, indicating overwintering. Field observations in Zurich also showed that Arabidopsis bore flowers and fruits throughout spring and summer in addition to the overwintering cohorts (Sato et al. 2019a). Thus, we studied both overwintering and non-overwintering cohorts. The UV-B dose during the growth of the non-overwintering cohorts was several times higher than that of the overwintering cohorts (**Supplementary Table S4**). In previous field studies of Arabidopsis, seedlings were typically transferred to the field to avoid asynchronous germination and high mortality at early developmental stages (Tian et al. 2003, Sato et al. 2019a). However, in such settings, the effect on seedlings cannot be examined. Thus, in addition to seedlings, we transferred seeds directly to the field to subject the whole life cycle to field environments. Although not all cohorts were successful (e.g. heavy rain soon after sowing washed seeds away, **Supplementary Table S2**), sufficient data were obtained for most cohorts.

No significant differences in fitness components (survival rate or fruit number; **Supplementary Fig. S2B** and **S2C**) or morphology (**Supplementary Fig. S2D**) were observed between *uvr8-1* mutant and L*er* WT in various field experiments in Zurich (**Supplementary Table S3**), regardless of season or transfer stage.

We also transferred seedlings to a high-elevation site in the Swiss Alps in the canton of Grisons (Mountain cohort). The maximum UV-B irradiation was higher in the mountain cohort than in Zurich (**Supplementary Table S4**). Under this environmental condition, *uvr8-1* showed a higher mortality rate in comparison to the L*er* WT in a non-overwintering cohort (*P* = 0.002, **Supplementary Fig. S2B, Table S3**). Unfortunately, other fitness traits could not be assessed due to extensive herbivory damage that occurred after bolting on all plants.

### Double mutants of *UVR8* and *CRY1* show severe defects under field conditions

**Fig. 2:**
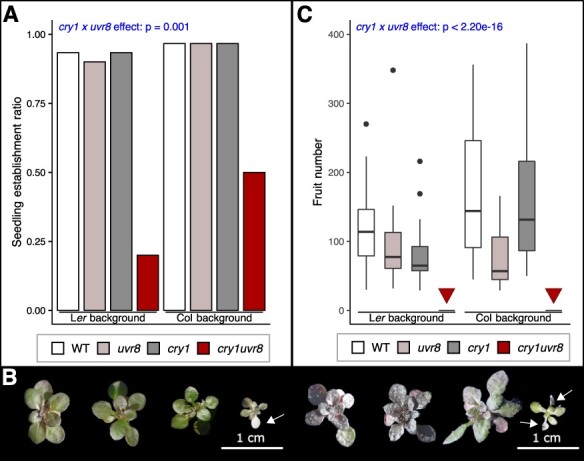
Reduced fitness of *cry1 uvr8* double mutants under field conditions. (A) Histogram of plant establishment success ratio in overwintering cohort 3 after approximately 4 months (123 d) in the field, with 1.00 being that all seeds grew to plants. Thirty replicates were analyzed for each genotype. Genotypes from left to right, as plotted: WT (L*er*), *uvr8* (*uvr8-1*), *cry1* (*hy4-2.23N*), *cry1uvr8* (*hy4-2.23N uvr8-1*), WT (Col-0), *uvr8* (*uvr8-19*), *cry1* (*cry1-304*) and *cry1uvr8* (*cry1-304 uvr8-19*). The significance level of the *cry1* × *uvr8* effect (statistical interaction) is given at the top of the plot. (B) Representative images of plants grown in overwintering cohort 3 for approximately 4 months (123 d) after seed transfer. Genotypes shown from left to right: WT (L*er*), *uvr8* (*uvr8-1*), *cry1* (*hy4-2.23N*), *cry1uvr8* (*hy4-2.23N uvr8-1*), WT (Col-0), *uvr8* (*uvr8-19*), *cry1* (*cry1-304*) and *cry1uvr8* (*cry1-304 uvr8-19*). The scale bar length is 1 cm, as indicated. White arrows indicate chlorotic leaves, which are only present on *cry1 uvr8* double mutants. (C) Boxplots of fruit number after 132 d under open-field conditions of overwintering cohort 3. Four genotypes of two different backgrounds (left: L*er*, right: Col-0) were included in this experiment. Genotypes and number of replicates (from left to right, as plotted): WT (L*er*), *n* = 16; *uvr8* (*uvr8-1*), *n* = 16; *cry1* (*hy4-2.23N*), *n* = 16; *cry1 uvr8* (hy4-2.23N uvr8-1), *n* = 16; WT (Col-0), *n* = 19; *uvr8* (uvr8-19), *n* = 20; *cry1* (*cry1-304*), *n* = 20; *cry1 uvr8* (*cry1-304 uvr8-19*), *n* = 20. Bars indicate the median, and boxes indicate the interquartile range. Whiskers extend to the most extreme data point that is not more than 1.5 times the interquartile range from the box, and outliers are indicated with dots. Red triangles indicate that no *cry1 uvr8* plants survived until fruit set. The significance level of the *cry1* × *uvr8* effect (statistical interaction) is given at the top of the plot. Further fitness components for plants grown in field environments with natural solar irradiation are presented in **Supplementary Fig. S2**.

To test for potential functional redundancy between UVR8 and cry1 in the field, we grew WT, *cry1, uvr8* and *cry1 uvr8* plants (in two different backgrounds, L*er* and Col; **Supplementary Table S1**) over two seasons. Our statistical analysis was centered on whether *cry1 uvr8* exhibited more severe defects in fitness components than the addition of the single mutant defects would explain. This was tested by including an interaction term (statistical interaction) between *uvr8* (functional or nonfunctional) and *cry1* (functional or nonfunctional), integrating data of the two backgrounds (**Supplementary Table S3**). We examined an overwintering cohort, in which plants were exposed to field conditions from the seed stage (**Fig. 2**, overwintering cohort 3). We found that *cry1 uvr8* plants had strongly reduced seedling establishment (statistical interaction, *cry1* × *uvr8*, *P* = 0.001, incorporating both accessions, the same below; **Fig. 2A**, **Supplementary Table S3**) and growth (**Fig. 2B**). Many of the leaves of the double mutants showed yellowing and eventually chlorosis (arrows in **Fig. 2B**). After 123 d (approximately 4 months), surviving plants of this genotype had severely reduced aboveground biomass (fresh weight) (*cry1* × *uvr8*, *P* = 5.54 × 10^−5^, **Supplementary Fig. S2E, Table S3**), whereas after 132 d, all *cry1 uvr8* plants had died (**Supplementary Fig. S2F**) without developing fruits (*cry1* × *uvr8*, *P* < 2.20 × 10^−16^, **Fig. 2C**, **Supplementary Table S3**). These fitness component data suggest that negative effects associated with the double mutants are synergistic rather than additive. We also conducted an experiment by seedling transfer in a non-overwintering condition, where only a few replicates were measurable due to a dysfunction in the irrigation systems (non-overwintering cohort 3, **Supplementary Table S2**). Nonetheless, the interaction effect on inflorescence dry weight was similarly significant despite the small sample size (*cry1* × *uvr8*, *P* = 1.35 × 10^−5^, **Supplementary Fig. S3A**, right panel, **Supplementary Table S3**). After 45 d under field conditions, *cry1 uvr8* double mutants in Col background were visibly different (**Supplementary Fig. S2G**) and a significant interaction of *cry1* and *uvr8* effects on anthocyanin content was detected (*cry1* × *uvr8*, *P* = 2.59 × 10^−8^, **Supplementary Fig. S2G, Table S3**).

### UV causes the conditional *cry1 uvr8* lethality

**Fig. 3:**
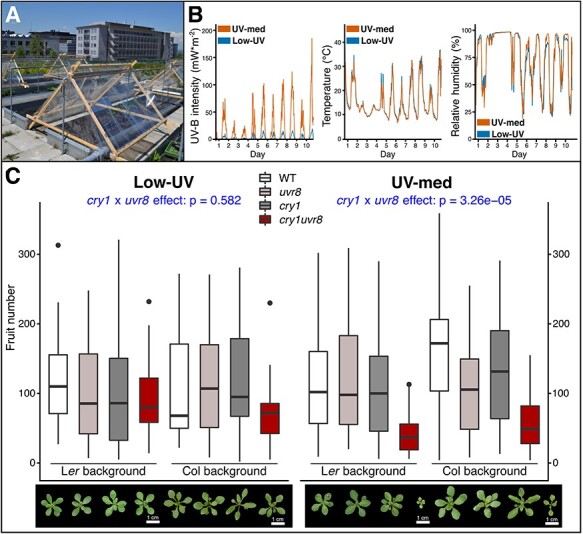
UV attenuation in the field. (A) Image of the set-up for the UV attenuation experiments at the Zurich common garden site. Tent structures are covered with different filtering films for Low-UV (Rosco #226) and UV-med (Rosco #130) conditions. (B) Line plots of environmental conditions in the UV attenuation environments Low-UV and UV-med in non-overwintering cohort 2. Values shown were recorded using a UV-Microlog (sglux) data logger. Three different environmental variables are shown for a 10-day interval from left to right: UV-B intensity (diode on the logger surface), temperature (sensor inside logging device) and relative humidity (external sensor). (C) Boxplot of fruit number for non-overwintering cohort 2 transferred to the Zurich common garden as seed on top. Plants were grown under two different UV conditions, left: Low-UV and right: UV-med. Dead plants that produced no seeds were not included in the graphical visualization. Four genotypes of two different backgrounds (left: L*er*, right: Col-0) were included. Genotypes and number of replicates (left to right as plotted, the first number refers to Low-UV, and the second to UV-med): WT (L*er*), *n* = 30|36; *uvr8* (*uvr8-1*), *n* = 34|28; *cry1* (*hy4-2.23N*), *n* = 40|28; *cry1uvr8* (*hy4-2.23N uvr8-1*), *n* = 43|41; WT (Col-0), *n* = 37|34; *uvr8* (uvr8-19), *n* = 33|36; *cry1* (*cry1-304*), *n* = 42|36 and *cry1uvr8* (*cry1-304 uvr8-19*), *n* = 39|41. Bars indicate the median, and boxes indicate the interquartile range. Whiskers extend to the most extreme data point that is not more than 1.5 times the interquartile range from the box, and outliers are indicated with dots. The significance level of the *cry1* × *uvr8* effect (statistical interaction) is given at the top of the plot. Representative images of plants grown in non-overwintering cohort 2 are depicted below. Low-UV (left) and UV-med (right) conditions 14 d after transfer to the field. Genotypes are in identical order to the histogram. Both double mutant genotypes grew visibly smaller than other genotypes. The scale bar length is 1 cm, as indicated. Further fruit-fitness components of field-grown UV attenuation cohorts are presented in **Supplementary Fig. S3**. Fitness components in Chamber-UV and +UV-B are presented in **Supplementary Fig S4**.

We next studied whether solar UV radiation is responsible for the severe defects of *cry1 uvr8* double mutants by growing plants under experimentally manipulated UV levels in the field. We constructed two types of UV screening tents with different levels of UV filtering: UV-blocking (Rosco #226) or UV-transmitting (Rosco #130) filter (**Fig. 3A**). We empirically validated the natural fluctuation of UV-B intensity, temperature and relative humidity (e.g. days 2 and 3 were rainy with lower UV-B intensity) (**Fig. 3B**). The UV-B dose under the tent with UV-blocking Rosco #226 filter was approximately 0.7% of natural UV-B irradiation (**Supplementary Table S4**), and so the condition was termed Low-UV. In the tent with the UV-transmitting Rosco #130 filter, our validation showed that the filter was unexpectedly not fully transparent to UV-B. Rather, the UV dose was approximately 25% of the daily UV-B dose in July, similar to the daily dose under winter conditions (**Supplementary Table S4**) in which double mutants did not die prematurely. The condition was termed UV-med. Our data show that temperature and relative humidity were comparable. In summary, the UV-B level was different between the two experimental conditions (Low-UV and UV-med) (**Fig. 3B**, **Supplementary Table S4**), whereas other conditions were similar.

We grew a non-overwintering cohort from seeds for a complete life cycle in the field (**Fig. 3C**; **Supplementary Table S2**; non-overwintering cohort 2). Two quantitative fitness components related to seed production were measured, i.e. fruit number and inflorescence dry weight, which were highly correlated (adjusted *R*^2^ ≥ 0.869; **Supplementary Fig. S3B**). In UV-med, *cry1 uvr8* double mutants showed reduced growth (**Fig. 3C**, right panel); however, they did not die prematurely, which enabled tissue sampling for subsequent RNA extraction and expression analysis (see later). Importantly, in Low-UV, *cry1 uvr8* growth was comparable to WT (**Fig. 3C**, left panel), thus demonstrating that solar UV is responsible for the *cry1 uvr8* defects, including the conditional lethality. Similar to the previous field experiments, we statistically tested the interaction effect of *UVR8* and *CRY1* on fitness components (**Fig. 3C**, **Supplementary Fig. S3C, Tables S3** and **S4**), which was significant in UV-med (fruit number: *P* = 3.26 × 10^−5^; inflorescence dry weight: *P* = 2.72 × 10^−4^) but not in Low-UV (fruit number: *P* = 0.582; inflorescence dry weight: *P* = 0.790). These results suggest that the synergistic defect of *UVR8* and *CRY1* is only detectable in the presence of considerable UV radiation. An effect of UV-B on fitness was additionally supported by a significant three-way interaction effect of *UVR8, CRY1* and UV-B condition (ANOVA; three-way interaction; fruit number: *P* = 0.037; inflorescence dry weight: *P* = 0.038). In addition, a similar pattern was observed in another small-scale non-overwintering cohort (non-overwintering cohort 3, **Supplementary Table S2**). The statistical interaction of *UVR8* and *CRY1* on their inflorescence dry weight was not significant in Low-UV, but significant in UV-med as well as under open conditions (**Supplementary Fig. S3A, Table S3**). These UV attenuation experiments corroborated that UV is the causal factor for *cry1 uvr8* growth defects.

Next, we experimentally supplemented UV-B under a laboratory growth chamber condition, as previously described (Arongaus et al. 2018). With the UV-B dose comparable to fall/winter in the field site in Zurich (**Supplementary Table S4**), *cry1 uvr8*, but not the respective single mutants, showed excess mortality (statistical interaction *P* < 2.2 × 10^−16^) and strongly impaired growth (**Supplementary Fig. S4A-C**), in agreement with previous findings in L*er* background (Tissot and Ulm 2020). By contrast, for plants grown under ‘UV-B exclusion’ as well as ‘Chamber-UV’ conditions in growth chambers, *cry1 uvr8* mutants did not show any obvious defects in survival or growth (**Supplementary Fig. S4B-E**). Combined with results from the UV attenuation experiments in the field, these data indicate that the reduced fitness of *cry1 uvr8* double mutants in the field is attributable to UV-B exposure.

### Gene expression profiles among field and laboratory conditions correspond to fitness component results

**Fig. 4:**
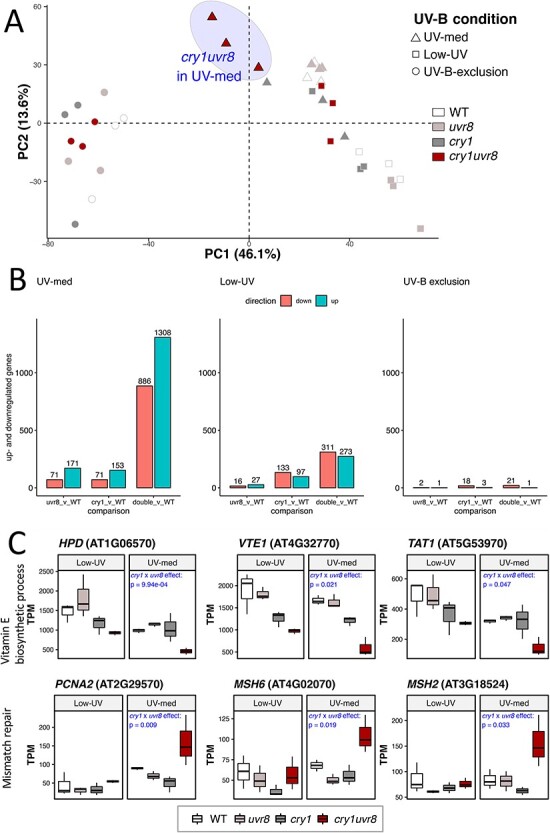
RNA-seq analysis. (A) PCA plot of the gene expression data of all 36 samples. The top two dimensions (*x*-axis = dimension 1, *y*-axis = dimension 2) are shown. The shape of the samples represents three different treatments, namely, UV-med (triangles), Low-UV (squares) and Chamber-UV-B exclusion (circles). The shadow of the shapes distinguishes the genotypes of the L*er* background: WT (blank), *uvr8* (*uvr8-1*, light shadow), *cry1* (*hy4-2.23N*, intermediate shadow) and *cry1uvr8* (*hy4-2.23N uvr8-1*, dark shadow). A light-blue shade highlights the differentiated samples of the *cry1uvr8* double mutant in UV-med. (B) Bar chart of DEGs from RNA-seq analyses. Three panels for each environment are shown, from left to right: ‘UV-med’, ‘Low-UV’ in the field and ‘UV-B exclusion’ in the chamber. For each panel, histograms for up- (blue) and downregulated (red) genes are shown for the expression of three genotypes compared to WT: a *UVR8* mutant (*uvr8-1*), a *CRY1* mutant (*hy4-2.23N*) and the double-mutant (*hy4-2.23N uvr8-1*). DEGs were identified with the criteria of FDR ≤ 0.05 and |logFC| > 1. (C) Boxplots for three genes of two GO terms with significant interaction effect of *cry1* × *uvr8*. The top row shows genes in GO:0010189 (vitamin E biosynthesis) with reduced expression levels in UV-med with significant statistical interaction (*cry1* × *uvr8*), and the bottom row shows genes in GO:0006298 (mismatch repair) with higher expression levels, respectively. Common gene names and identifiers are given at the top of corresponding plots. Low-UV and UV-med are shown in the left and right boxplots for each gene. The *y*-axis represents the gene expression in transcripts per million. The maximum values of the *y*-axis vary between the six genes. Three replicates were included for each genotype, genotypes from left to right: WT L*er, uvr8-1* (*uvr8*), *hy4-2.23N* (*cry1*) and *hy4-2.23N uvr8-1* (*cry1 uvr8*). The *P*-value of the *cry1* × *uvr8* effect (statistical interaction) is given at the top of each individual plot when it is significant. If no significance was found, no value is depicted.

We performed a transcriptome analysis to molecularly characterize the impairment of *cry1 uvr8* double mutants as well as that of the respective single mutants. For this, we examined the genome-wide expression patterns of 14-day-old plants grown from the seed stage under two field conditions (Low-UV and UV-med), as well as in a growth chamber in the absence of UV-B. Our experiment was designed to study gene expression profiles underlying UV acclimation and stress tolerance rather than short-term, acute responses, which has been done previously (Rai et al. 2020). We analyzed a total of 36 samples representing three biological replicates each of four L*er* genotypes (L*er, hy4-2.23N, uvr8-1* and *hy4-2.23N uvr8-1*; note that *hy4-2.23N* is a *cry1* mutant allele) grown under three conditions (UV-med and Low-UV in non-overwintering cohort 2 and UV-B exclusion in chamber cohort 6) (**Tables S5** and **S6**). We performed a principal component analysis (PCA) to examine the most influential factors on gene expression. The first two axes (principal components 1 and 2, i.e., PC1 and PC2) had a major effect on gene expression (46.1% and 13.6%, respectively, **Fig. 4A**). PC1 corresponds to the difference between the field conditions and the UV exclusion in a growth chamber, supporting a major difference between the regulated and field conditions. PC2 corresponds to the genotypic differences, driven by the separation of double mutants in UV-med.

We then identified differentially expressed genes (DEGs) of the two single mutants and a double mutant compared to the WT with a false discovery rate (FDR) lower than or equal to 0.05 and >2 fold change (**Fig. 4B**). Under the UV-B exclusion condition in a growth chamber, only very a few significantly up- or downregulated genes were found (**Fig. 4B**). Among the three mutants under the two field conditions, the double mutants under the UV-med conditions showed many more DEGs (886 and 1,308 genes were significantly down- and upregulated, respectively) (**Fig. 4B**). We conducted a Gene Ontology (GO) analysis of DEGs under the UV-med conditions (**Supplementary Tables S7** and **S8**). The GO of response to UV-B (GO:0010224) was enriched among downregulated genes of all three mutants as expected. The number of significant genes of this GO category was highest in the double mutant (**Supplementary Table S8**), which included *PYRIDOXINE BIOSYNTHESIS 1.3* (*PDX1.3*) encoding an enzyme in the vitamin B6 biosynthesis (Tambasco-Studart et al. 2005, Ristilä et al. 2011, Morales et al. 2013), *ELONGATED HYPOCOTYL 5* (*HY5*) encoding key bZIP transcriptional regulators of light response (Bobby A. Oyama et al. 1997, Ulm et al. 2004, Brown et al. 2005) and *REPRESSOR OF UV-B PHOTOMORPHOGENESIS 2* (*RUP2*) that is directly involved in a negative feedback loop regulating UVR8 activity (Gruber et al. 2010, Heijde and Ulm 2013).

Many DEGs in the double mutant in comparison with that in single mutants suggest that the expression of many genes was under synergistic, non-additive effects of *uvr8* and *cry1* mutations, but the DEG analysis cannot directly show which genes were under the synergistic effect. Thus, analogous to statistical interactions on fitness components described in previous sections, we next identified genes whose expression level shows significant statistical interaction effects of cry1 and UVR8. We found that as many as 1,438 genes showed a statistically significant interaction of *uvr8* and *cry1* mutations (FDR-adjusted *P* ≤ 0.05) under the UV-med condition (**Table S9**). This number was much lower in Low-UV (141 genes; **Supplementary Table S10**) and very low under the UV-B exclusion condition (five genes; **Supplementary Table S11**), supporting the synergy of UVR8 and cry1 in response to UV irradiation. **Fig. 4C** illustrates the decreased (**Fig. 4C**, upper) and increased (**Fig. 4C**, lower) expression, respectively, in the double mutants under the UV-med condition (right panels). It also shows non-significant differences in their expression under the Low-UV condition (left panels) (**Supplementary Tables S12**). Among the 1,438 genes with significant interaction in UV-med, 513 and 520 genes showed a >2-fold decrease or increase in expression, respectively. From these 513 genes with reduced expression, 150 GO terms were significantly enriched (**Supplementary Table S13**). Response to light stimulus (GO:0009416), specifically response to UV-B (GO:0010224) and blue light signaling pathway (GO:0009785), was enriched, along with pathways that are directly affected by cry1 and UVR8 function, e.g. flavonoid biosynthetic process (GO:0009813) and regulation of photomorphogenesis (GO:0010099). Categories related to photosynthesis and response to oxidative stress were also enriched, including response to oxidative stress (GO:0006979) and the vitamin E biosynthetic process (GO:0010189) (**Fig. 4C**, upper, **Supplementary Table S12**). As expected, most of these GO categories were also significant in the GO analysis of downregulated genes in the double mutants described earlier (**Supplementary Table S8**). Next, among the 520 genes with increased expression, 180 GO categories were enriched (**Table S14**). DNA damage repair terms were enriched, e.g. mismatch repair (GO:0006298) (**Fig. 4C**, lower, **Table S12**), double strand break repair (GO:0006302) or recombinational repair (GO:0000725) (see the Discussion section).

## Discussion

### Fitness consequence of UVR8-mediated responses

*Arabidopsis* studies have shown that mutant plants lacking stress tolerance sometimes show altered fitness (growth and reproduction) compared with WTs in the absence of the stress factor compared to those in its presence. Such intrinsic fitness costs were reported using Arabidopsis single-gene mutants impaired in resistance to disease (Tian et al. 2003), herbivore treatment (Züst et al. 2011, Sato et al. 2019a) and herbicide treatment (Purrington and Bergelson 1999, Roux et al. 2004). For example, *csr1-1* and *ixr1-2* mutants, which gain resistance to acetolactate synthase or cellulose synthase inhibitors, exhibit 36–44% reduction in total fruit production in the absence of these herbicides (Roux et al. 2004). In a natural population, *A. halleri glabra1* (*gl1*) trichome-less mutants were shown to produce more seeds than trichome-producing (hairy) plants, but only when herbivores were reduced by insecticide treatment (Kawagoe et al. 2011). In the presence of herbivore stress, the fitness of *A. halleri* plants with or without trichomes was equivalent, suggesting that the benefit of trichomes as herbivore defense and the associated fitness cost offset each other in the presence of herbivore stress (Kawagoe et al. 2011). In the absence of the herbivores in a chamber, *gl1* mutants and several mutants of *A. thaliana* defective in glucosinolate biosynthesis showed 10–18% increased growth rates (Züst et al. 2011). These studies of stress responses suggest a trade-off of benefit and negative pleiotropic effects on fitness components due to the alteration of physiological processes. Similar to the aforementioned examples, we report here the intrinsic cost of a response pathway resulting in abiotic stress tolerance, namely, the UVR8-mediated UV-B response. With UV-B levels under fluorescent white light tubes in a standard growth chamber corresponding to only approximately 1.5% of natural UV-B irradiation, the fitness components of *uvr8* mutants in different Arabidopsis accessions showed higher values than the corresponding WT plants. This implies that UVR8-mediated UV-B responses were induced, but UV-B damage was very low if not absent under these conditions. Indeed, under the UV-B exclusion conditions, no significant differences in fitness components of WTs and *uvr8* mutants were detected, indicating the inductive nature of UV-B responses. The estimated fitness cost (12–72%, **Fig. 1**, **Supplementary Fig. S1**) is comparable to previous studies and depended on measured fitness components and on growth conditions owing to the inducible nature of the response. By contrast, in the presence of a more significant stressor, i.e. sunlight in Zurich, no significant difference in fitness components was detected. We deduce that the offset of the benefit and fitness cost underlies the similar fitness of WT and mutants in our experiments as well as the lack of clear phenotypes in previous field studies (Morales et al. 2013, Coffey et al. 2017, Santhanam et al. 2017). In contrast to naturally occurring mutations in herbicide resistance and herbivore defense, *UVR8* is generally highly conserved (Fernández et al. 2016, Tilbrook et al. 2016, Han et al. 2019, Podolec et al. 2021a), suggesting that its benefit would be higher than its cost in general.

Although we did observe excess mortality of *uvr8* mutants in a small-scale experiment at an alpine site, this finding remains to be repeated in future field experiments using a broad range of altitudinal locations. Moreover, we would like to note that the alpine site data alone cannot establish if UV-B irradiation had the causal lethal effect in the mountain cohort. The amount of UV irradiation in the alpine environment is subject to large fluctuations and may not have been greater than that in the lowland (Blumthaler 2012). In addition, altitudinal gradients in irradiation, temperature, rainfall and others are correlated (Körner 2003). Therefore, larger-scale studies encompassing diverse environments would be needed to get an even broader picture of the *UVR8* gene function *in natura*.

### Synergistic effect of *CRY1* and *UVR8* mutations on fitness components shown by statistical interactions

The interaction in the downstream cascade of different photoreceptors is of major interest in photobiology. Recent studies suggested a common mechanism of UVR8- and cryptochrome-mediated inhibition of COP1 (Favory et al. 2009, Podolec and Ulm 2018, Lau et al. 2019, Ponnu et al. 2019, Podolec et al. 2021a), and a *cry1 cry2 uvr8* triple mutant showed lethality under UV-B under natural conditions (Rai et al. 2019). By growing single and double mutants in diverse experimental settings, we here provide substantial evidence that functional alleles of *UVR8* and *CRY1* synergistically prevent severe defects under UV-B under field conditions.

We found parallel evidence for fitness increase/decrease among different background accessions with a single or double mutation of *UVR8* and *CRY1*. Such consistent results under diverse environmental conditions strongly suggest roles for these two genes in plant adaptation to sunlight *in natura*. Significant statistical interaction indicated that the loss-of-function effects of *UVR8* and *CRY1* on fitness components were synergistic. In addition, when double mutants were grown from seeds, conditional synthetic lethality was observed. Similar results were obtained in both overwintering and non-overwintering cohorts in Zurich. The use of UV filters in the field restored normal plant growth in *cry1 uvr8*, further confirming that their defects are caused by UV, in agreement with a previous study of *cry1 cry2 uvr8* triple mutants in the field (Rai et al. 2019). Moreover, the growth of plants in a chamber with supplemental UV-B recapitulated the elevated sensitivity of *cry1 uvr8* double mutants used in this work when compared to the respective single mutants and WT, in agreement with previous findings (Tissot and Ulm 2020).

Cryptochromes evolved before the split of plants and animals and may have played an ancestral role in blue/UV-A light sensing and response. *UVR8* is thought to have originated later, and its function was shown to be conserved between green algae and angiosperms (Rizzini et al. 2011, Allorent et al. 2016, Fernández et al. 2016, Tilbrook et al. 2016, Han et al. 2019, Podolec et al. 2021a). Our data strongly support that *UVR8* and *CRY1* are synergistically required for plant survival under sunlight (Rai et al. 2019, Tissot and Ulm 2020).

### Synergistic effect of *CRY1* and *UVR8* mutations on the expression of genes responsible for UV response and DNA integrity

To test the statistical interaction of *uvr8* and *cry1* mutations at the gene expression level similar to fitness-related traits, we obtained RNA-seq data of the *uvr8 cry1* double mutant together with those of single mutants exposed to long-term field conditions. A previous study reported RNA-seq data of *UVR8* and *CRY* mutants separately (Rai et al. 2020). Here, we found that the *cry1 uvr8* double mutant showed several times more DEGs than each mutant in the UV-containing (UV-med) condition (**Fig. 4b**).

The RNA-seq data of the *uvr8 cry1* double mutants allowed us to detect significant statistical interaction of *uvr8* and *cry1* mutations at the gene expression level similar to the analysis of the fitness-related traits. Among the genes with significant interaction under UV-med conditions, 513 and 520 genes were >2-fold up- and downregulated, respectively. GO analysis of the latter suggested biological processes of mainly three groups: light response, photosynthesis and oxidative stress. Among these terms were response to UV-B (GO:0010224), blue light signaling pathway (GO:0009785), flavonoid biosynthesis (GO:0009813) and regulation of photomorphogenesis (GO:0010099). Photosynthetic machinery is also susceptible to high-light stress, especially UV (Takahashi et al. 2010, Lütz and Seidlitz 2012, Demarsy et al. 2018). An important role in maintaining photosynthetic function under such conditions is attributed to certain protective compounds, such as tocopherol (Vitamin E), for which deficits lead to photooxidation and chlorosis (Havaux et al. 2005, Ksas et al. 2015, Miret and Munné-Bosch 2015). Consistent with chlorotic leaves found in double mutants (**Fig. 2B**), the expression levels of vitamin E biosynthesis genes (**Fig. 4B**, upper panels, **Supplementary Table S12**) showed significant statistical interaction and were also reduced in *cry1 uvr8* double mutants (**Supplementary Table S7**).

The 520 genes that showed increased gene expression and statistical interaction in UV-med were mainly enriched for methylation and DNA repair–related terms, such as mismatch repair (GO:0006298). The upregulation of two DNA mismatch repair protein genes (*MSH2* and *MSH6*) and the *PCNA2* gene (**Fig. 4B**, lower panels, **Supplementary Table S12**) attributes a potential role to UVR8 and cry1 in the maintenance of DNA integrity under solar UV(-B). We speculate that the impaired UV responses described earlier resulted in DNA damage following UV irradiation, and thus, DNA repair pathways may have been upregulated to compensate for the UV-induced damage.

Besides the RNA-seq data of the *uvr8 cry1* double mutant, a unique feature of our RNA-seq data is the long-term growth of mutants under field conditions from the seed stage. Previously, a quantitative PCR of nine UV- and blue light–responsive marker genes was conducted following 17 d of exposure, but the response was mostly non-significant in a *uvr8* mutant, a *cry1 cry2* double mutant and a *cry1 cry2 uvr8* triple mutant (Rai et al. 2019). By contrast, our RNA-seq analysis identified more than a thousand DEGs in the *uvr8 cry1* double mutant. The DEGs include genes responsible for diverse processes including DNA repair in addition to genes that are known to be induced immediately by UV-B. This highlights the importance of studying the long-term effect of field conditions on genome-wide gene expression patterns.

### Conclusion

Our data highlight the complex nature of light responses throughout plant life stages and the importance of combining field and laboratory experiments. Using a genetically tractable species such as *Arabidopsis thaliana*, we were able to add to the understanding of the molecular foundation of abiotic stress responses in plants. To test the ecological relevance of cry1 and UVR8, we applied a dual method: assessment of the quantitative fitness and gene expression variation of different genotypes, including single and double mutants in a factorial design. This approach enabled us to gain insight into the interaction effects of two important photoreceptors perceiving blue light and UV-B, respectively, crucial for UV-B tolerance in the field. Thus, our study showcases the value of combining mutant analyses with ecological functional genomics in understanding the molecular basis of plant environmental response *in natura*.

## Materials and Methods

### Genotype information

*Arabidopsis thaliana* mutants *uvr8-1* (Kliebenstein et al. 2002), *hy4-2.23N* (Ahmad and Cashmore 1993) and *hy4-2.23N uvr8-1* (Tissot and Ulm 2020) are in the L*er* background; *uvr8-19* (Podolec et al. 2021b), *cry1-304* (Mockler et al. 1999) and *cry1-304 uvr8-19* (Podolec et al. 2021b) in Columbia-0 (Col-0) and *uvr8-7* (Favory et al. 2009) in Ws. See **Supplementary Table S1** for further details on these lines.

### Experimental conditions

We prepared common gardens in Zurich at the Irchel Campus of the University of Zurich (47°23’46.1” N, 8°33’04” E, 500 m altitude) and Calanda, Grisons (46°53’16.1” N, 9°29’21.6” E, 2000 m altitude, mountain cohort), as well as growth chambers. The Calanda site was kindly provided by the Gemeinde Haldenstein (Switzerland) and managed and permitted by the Plant Ecological Genetics Group in the Institute of Integrative Biology of ETH Zurich (Switzerland).

For chamber experiments, we used custom-built growth chambers (Kälte 3000) equipped with Regent ‘Easy 5 Cool White’ (FDH-39W) and Regent ‘!GroLux’ (FDH-39W) batten luminaires in a 2:1 ratio. We used two different conditions for experiments in growth chambers. Long-day conditions with 16 h light (22°C, 60% relative humidity, 120–160 µE light) and 8 h darkness (20°C, 60% relative humidity) were used for plant pre-treatment as well as in Chamber-UV, UV-B exclusion and +UV-B conditions. Short-day conditions were 8 h light (18°C, 60% relative humidity, 120–160 µE light) and 16 h darkness (16°C, 60% relative humidity).

Each growth experiment was with multiple genotypes arranged in a random block design. **Supplementary Table S2** shows the experimental duration, the initial number of transferred individuals per genotype, the plant growth stage at transfer for each experiment, the UV-B levels, whether the experiment was disrupted before data could be acquired and the corresponding figures. The set-up for growth chamber experiments closely resembled that of field experiments, with the addition of mild insecticide (50 g/l RAVANE 50, Schneiter AGRO, Seon, Switzerland) and fungicide treatments (1 g/l Thiovit Jet, Maag Garden, Dielsdorf, Switzerland) every 14 d.

Environmental data were recorded using UV-Microlog miniature data loggers (sglux¸ https://sglux.de/). These loggers are weather-resistant and provide logging function of three independent environmental variables over longer time intervals. The loggers were equipped with a UV-B diode (TOCON_E_OEM, sglux) and an integrated temperature and an external humidity sensor (relative humidity in percent). The output shown in this study for the UV-Microlog is the erythemally weighted UV-B intensity in mW m^−2^.

The loggers were used to record environmental data for several days, performed at least once for all the field and chamber conditions. In non-overwintering cohort 2, three loggers were used in parallel to compare UV attenuation with unfiltered UV-B levels (**Supplementary Table S4**). The data loggers were placed on even ground directly in the compartments.

### Plant material

Plants were transferred to the field either as seeds or as seedlings at the five-leaf rosette stage, respectively (**Supplementary Table S2**). We directly transferred seeds to the field to investigate full life cycles, although experiments were occasionally destroyed owing to the vulnerability of young seedlings to natural phenomena such as heavy rain or drought. Seedlings, therefore, were brought to the field for some of the experiments, as is commonly done in Arabidopsis field studies (Tian et al. 2003, Sato et al. 2019a) as a backup for measurements in case seed–derived plants were lost.

For the transfer at the seed stage, we stratified seeds by putting them on 0.8% agar plates with 1/2 Murashige and Skoog (MS) medium or in Eppendorf tubes with tap water at 4°C in darkness for up to 72 h. Three to five seeds were then transferred to the surface of watered standard soil (Einheitserde) in plastic pots (8 × 8 × 7 cm). The pots were kept at room temperature overnight and then transferred to the field. After germination and before the leaves of plants growing in the same pot began to touch, thinning was performed in all experiments by cutting off and removing aboveground plant parts to obtain one plant per pot.

As preparations for the transfer of plants to the field at the five-leaf stage, seeds were sown on 0.8% agar plates with 1/2 MS medium. After 72 h at 4°C in darkness, plates were transferred to growth chambers with long-day conditions to induce germination. Germinated plants were then transferred to soil (Einheitserde) in plastic pots (8 × 8 × 7 cm) and kept in growth chambers under short-day conditions to avoid early flowering onset until plants reached a five-leaf stage. For acclimation, the seedlings in the plastic pots were transferred to shaded places in the common garden 24–48 h before placing them in the compartments.

Plants for growth chamber experiments were prepared in the same way as plants for field experiments transferred at the seed stage until potting. The potted plants were then placed in one of the chamber conditions (Chamber-UV, UV-B exclusion and +UV-B).

### Field site growth experiments

In the Zurich common garden, each compartment was filled with a 15-cm layer of ‘Rasenerde’ (top-dressing) and enclosed by a slug barrier. We watered each compartment automatically (three fine-spraying valves per compartment, 10 min duration, set at 05:00 and 21:00, respectively) between March and November and manually between November and March when the water supply was turned off to avoid frost damage to the water supply system. Pots were arranged at least 10 cm from the edges of the compartments and distributed with at least a 2-cm gap between pots.

To test the influence of UV on *uvr8* and *cry1* mutants, we prepared a total of six tents with wooden frames covered with UV-blocking (Rosco #226) or UV-transmitting (Rosco #130) filters (*n* = 3 for each filter type). The experiments were conducted in non-overwintering cohorts to avoid breakage and snow cover of UV filters by winter conditions.

At the high-elevation field site (Mountain cohort, 2000 m), a 1 × 2 m compartment without enclosure was prepared. Ten centimeters of the topsoil layer was exchanged with standard soil (Top-Dressing ‘Rasenerde’), and metal wire and fleece were embedded 10 cm below the soil surface of the compartment to avoid disturbances by fossorial animals.

### Chamber growth experiments

In the chamber experiments, we used the normal Chamber-UV (under fluorescent lamps, as described earlier). In addition, UV-B exclusion conditions were established by applying the UV-blocking filter film (Rosco #226, **Fig. S4E**) and supplemental UV-B in +UV-B was added from Philips TL20W/01RS narrowband UV-B tubes (Arongaus et al. 2018). Pots were placed in the corresponding chamber conditions and watered manually every 2–3 d. Water levels were controlled to be at a height of ca. 1.5 cm after watering. During flowering, plant floral stems were bound to a wooden stick in the center of the pot to avoid contact between flower stems from different individuals.

### Plant trait measurements

We measured fitness components of survival and reproductive output (fruit number, inflorescence dry weight; and in chamber cohort 2, fruit length and seeds per fruit were additionally assessed). Furthermore, biomass (fresh weight of aboveground plant parts) in overwintering cohort 3 as well as in chamber cohort 4 was assessed. In non-overwintering cohort 3, anthocyanin accumulation was measured. Survival was recorded at the time of harvest as the presence/absence of a plant in each pot. In addition, seedling establishment (after germination success) was measured in overwintering cohort 3 to infer the survival of germinated plants. These individuals were then monitored for their survival until flowering onset.

To assess biomass, rosettes were harvested and immediately placed in liquid nitrogen to avoid drying. After collection, the frozen plant material was weighed on a precision balance.

Reproductive output in chamber experiments was evaluated by counting the fruit number (siliques). Fruit number was assessed in plants of chamber cohorts 1–3, overwintering cohort 1 and overwintering cohort 3 as well as non-overwintering cohorts 1–2. In addition, in non-overwintering cohort 2–3, aerial plant parts above rosette leaves were harvested together after primary and secondary inflorescences ceased flower production, dried at 60°C for at least 24 h and then weighed on a precision balance to determine the inflorescence dry weight. Inflorescence dry weight and fruit number were highly correlated (**Fig. S3B**), and therefore, only the former was measured for non-overwintering cohort 3, in which the plants grew large and the fruit number was very high. The absence of plants at the time of harvest was recorded as zero count, and statistical analysis was performed without zeros for a more conservative analysis, except for fruit number in the case of overwintering cohort 3, where we performed an analysis with zeros, as no surviving double-mutant plants were observed (see the statistical analysis section).

### Transcriptomic analysis

#### RNA extraction

Samples of the L*er* genotypes were collected following 2 weeks (14 d) growth under field (UV-med and Low-UV in non-overwintering cohort 2) and chamber (UV-B exclusion in chamber cohort 6) conditions. We sampled rosette leaves between 11:00 and 14:00 to avoid gene expression fluctuation caused by the effects of diurnal rhythms. The material was collected in 1.5-ml vials, directly transferred to liquid nitrogen and then stored at −80°C until RNA extraction. We used the RNeasy Plant Mini Kit (Qiagen) for RNA extraction following the manufacturer’s protocol without DNase treatment. RNA quantity was measured using a Qubit 2.0 (Thermofisher Scientific) and then diluted to 25 ng/µl per sample.

#### Library preparation and sequencing.

Total RNA (500 ng per sample) was used to synthesize libraries using a TruSeq Stranded mRNA Kit v2 (Illumina). Cluster generation was performed at the Functional Genomics Center Zurich (Switzerland) using 10 mM of pooled normalized libraries on the cBOT with a TruSeq PE Cluster Kit v3-cBot-HS (Illumina). Subsequently, Illumina HiSeq 4000 sequencing was performed to generate the reads.

#### Data processing.

The data analysis framework SUSHI (Hatakeyama et al. 2016) was employed to process raw reads. Standard settings implemented in SUSHI were used for RNA-seq data processing. Data analysis was performed according to the following steps:

For quality analysis, ‘FastQC’ v 0.11 (Andrews 2010) was used. To align the reads to the Araport 11 Arabidopsis reference genome (Cheng et al. 2017), ‘STAR’ (Dobin et al. 2013) was used. We then assigned mapped reads to genomic features with FeatureCounts and used CountQC, implemented in Qualimap (García-Alcalde et al. 2012) for quality control after counting.

Further analysis was performed in RStudio v1.0.143 implementing R v3.3.3 and above (http://www.r-project.org/). Packages ggplot2 (Wickham 2016) and ggpubr (Kassambara 2019) were used to create graphics. Mapped and quantified reads were used for a PCA on all genes to identify the most contributing dimensions, employing packages DEseq2 (Love et al. 2014), factoextra (Kassambara and Mundt 2020) and FactoMineR (Lê et al. 2008).

Differential gene expression analysis was conducted using DESeq2. Our goal was to identify gene–gene interaction effects within and across UV attenuation treatments.

To estimate statistical power, we fitted two fully factorial models. Model 1 included factors for gene function of *CRY1* and *UVR8*, treatment and statistically significant interactions within and between genes and treatment. We increased statistical power by reducing complexity in model 2, which was based on by-treatment subsets of data, separating Low-UV from UV-med. GO enrichment analysis was performed on this set of genes with topGO (Alexa and Rahnenführer 2021) using the elim algorithm. To reduce redundancy due to GO term hierarchy, the identified GO categories were filtered to those categories with at least 10 and less than 1000 annotations.

### Relative anthocyanin accumulation

Leaf anthocyanin content was determined by spectrophotometry according to an adjusted method (Schmidt and Mohr 1981). Pre-weighed Arabidopsis leaf tissue was placed in 800 µl of extraction buffer (2-propanol:HCl:H_2_O in 18:1:81% by volume), boiled for 3 min and then kept at room temperature in darkness overnight. The samples were then centrifuged at 10000 rpm for 2 min, and the absorption of extracted anthocyanin was measured at 535 nm and 650 nm. Leaf anthocyanin content was then calculated by subtracting the absorption at 650 nm from that at 535 nm and dividing this by fresh weight (*y* = (*A*_535_ − A_650_)/fresh weight). This anthocyanin measurement was performed only for field-grown plants because *Arabidopsis* species are known to accumulate visible and high levels of anthocyanin pigments under field conditions (Akiyama et al. 2023).

### Statistical analysis

All statistical analyses were performed in R v3.3.3 and above (http://www.r-project.org/). In box plots, bars indicate the median and boxes indicate the interquartile range. Whiskers extend to the most extreme data point that is not more than 1.5 times the interquartile range from the box, and outliers are indicated with dots.

Explanatory variables consisted of *uvr8_*mutant (fixed effect), *cry1_*mutant (fixed effect), treatment (fixed effect), background (random effect) and block (random effect). The variables *uvr8_*mutant and *cry1_*mutant indicate gene functions. For each variable, whether the following mutations *uvr8-1, uvr8-7, uvr8-19, hy4-2.23N, cry1-304, hy4-2.23N uvr8-1* or *cry1-304 uvr8-19* existed was scored as yes or no. See **Supplementary Table S15** for an overview of scoring. Treatment was defined only for UV manipulation experiments in the field. This variable consisted of two levels created by the different filter types: UV-med or Low-UV. Background consisted of L*er*, Col or Ws. Within each block, one individual of a combination of *uvr8*_mutant, *cry1*_mutant, treatment and background was assigned, except for overwintering cohort 1, overwintering cohort 2, non-overwintering cohort 1 and mountain cohort, where a random design across each compartment was applied.

Using these variables, we built different models depending on the purpose and set-up of the experiment. To test the effect of the mutant genotype on fitness components in chamber experiments, we included *uvr8*_mutant as explanatory variables in the model. In chamber and field experiments using *cry1_*mutant, we built a model for each of the summer and winter cohorts with *uvr8*_mutant and *cry1*_mutant as explanatory variables in order to test the effect of mutants on fitness components. In field experiments, we also examined the effect of the interaction between *uvr8*_mutant and *cry1_*mutant by adding an interaction term in the model. To test the effects of mutants, UV and interaction thereof on fitness components in the UV manipulation experiment in the field, we built a model with *uvr8*_mutant, *cry1_*mutant, treatment (referring to the UV conditions Low-UV and UV-med), two-way interactions (*uvr8*_mutant × *cry1*_mutant, *uvr8*_mutant × treatment, and *cry1*_mutant × treatment) and a three-way interaction (*uvr8*_mutant × *cry1*_mutant × treatment) as explanatory variables in the model. In all models, we included background and block as explanatory variables, when applicable.

We adopted a linear model (LM) framework that was suitable for binary, count and continuous traits with additional sources of trait variation considered (Faraway 2016). Survival data were scored binary, and therefore, generalized LMs (GLMs) or generalized linear-mixed models (GLMM) with binomial distribution were built. In the case of overwintering cohort 3, data showed complete separation, i.e. the range of values of a response variable for one group of an explanatory variable did not overlap with that of an(other) group(s) of the same explanatory variable. In this case, no model would fit the data properly. Therefore, we transformed the data by adding a count of one (1) to each individual observation before fitting a model.

For non-survival data (i.e. biomass, anthocyanin, fruit number, inflorescence dry weight, fruit length and fruit seed number), we used LMs and linear-mixed models (LMM) when normality could be assumed by histograms and univariate Shapiro–Wilk normality tests. Otherwise, we used GLM or GLMM. We used R packages ‘stats’, ‘glmmTMB v 0.2.3’ (Brooks et al. 2017) and ‘lme4 v 1.1’ (Bates et al. 2015).

Analysis with GLM and GLMM was done in the following steps. First, we built three models with different distributions, i.e. Poisson, negative binomial as well as quasi-Poisson (or type I). Models that failed to converge or that converged with warnings were excluded from further steps. When at least two models were applicable, we determined the best model using the AICtab function of the package ‘bblme v1.0.20’. The model with the lowest likelihood ratio score was considered the best model. The best model was then examined for the fit of data by visually examining simulated standard residual plots (≥250 simulations per model to reduce stochastic errors), by the one-sample Kolmogorov–Smirnov test and by the outlier test using the package ‘dHARMA v0.2.3’ (Hartig 2018). When the best model did not appropriately fit the results, we built new models with Poisson, negative binomial or quasi-Poisson distribution with zero-inflation parameters using the package ‘glmmTMB’, with zero-inflation parameters applying to all observations (*z_i_* = ∼1 or *z_i_* = ∼.) or absences varying by specific factors (e.g. treatment, gene functions). These models were evaluated using the same steps as earlier. In the case of LM and LMM, we generally built a single model including all random factors and then continued with examining the best model for the fit of data as earlier.

Once the best model was identified, we tested the significance of the fixed effect(s) on the response variable by conducting an analysis of variance using the function ANOVA in the package ‘car v3.0-2’. When one or more interaction terms were present in the model, we used a type-III Wald chi-squared test. Otherwise, we used a type-II Wald chi-squared test (**Table S3**).

## Supplementary Material

pcad113_Supp

## Data Availability

Raw sequence reads used for RNA-seq analysis in this study are available at the DNA databank of Japan (SAMD00199169–SAMD00199240).
