# Supplementary material for: UV RESISTANCE LOCUS 8–Mediated UV-B Response Is Required Alongside CRYPTOCHROME 1 for Plant Survival in Sunlight under Field Conditions
Source: Plant Cell Physiol. 2023 Sep 27;65(1):35–48. doi: 10.1093/pcp/pcad113 (PMC10799719; doi:10.1093/pcp/pcad113)
Supplement: pcad113_Supp [file pcad113_supp.zip › suppl_data.zip!/suppl_data/pcp-2023-e-00210-File006.docx]

**Figure Supplements**

**Fig. S1: Fitness components of *uvr8* mutants in growth chamber experiments.**

**A**. Boxplot of fruit number in *uvr8* null mutants in two backgrounds, comparing wild type (WT, white) and *uvr8*-mutant (*uvr8*, shaded) plants in independent experiments in a standard growth chamber under white light fluorescent tubes containing very low levels of UV-B (“Chamber-UV”), in addition to the experiment depicted in Fig.1. Two experiments were performed in the L*er* background (Chamber cohort 2, Chamber cohort 3) and one comparison Ws (Chamber cohort 2), respectively. Genotypes used and number of replicates (left to right, as plotted): WT (L*er*), n = 8; *uvr8* (*uvr8-1*), n = 7; WT (L*er*), n = 6; *uvr8* (*uvr8-1*), n = 6; WT (Ws), n = 8; *uvr8* (*uvr8-7*), n = 8. Significance levels indicated by asterisks are based on Analysis of Variance of linear models explaining fruit number by *UVR8* function for each subset of data by background; (**p < 0.01; *p < 0.05). The p-value for the UVR8 function effect over both backgrounds is given in blue at the top of the plot.

**B,C**. Additional fruit traits measured in “Chamber-UV” within Chamber cohort 2. Boxplot of fruit length (**B**) and seeds per silique (fruit) (**C**) for the wild type (WT) and *uvr8* mutant of two backgrounds (L*er*, Ws). Five siliques per plant were measured for both traits. Genotypes used and number of replicates from left to right, as plotted: WT (L*er*) n = 8; *uvr8* (*uvr8-1*), n = 7; WT (Ws), n = 8; *uvr8* (*uvr8-7*), n = 8.

**D**. Boxplot of fresh weight (aboveground biomass) after 21 days in “Chamber-UV” conditions (Chamber cohort 4). Asterisks represent p-value from generalized linear model explaining fresh weight, in separate analysis by background. P-values from left to right, as plotted: p = 0.014, p = 0.049. Genotypes and number of replicates from left to right, as plotted: L*er*, n = 9; *uvr8-1*, n = 6; Col-0, n = 6; *uvr8-19*, n = 7.

**E.** Boxplot of the fruit number (siliques per plant) for *uvr8-1* (*uvr8*) and wild type L*er* (WT) grown in UV-B-exclusion (Chamber cohort 5) chamber experiments, fourteen replicates per genotype (n = 14)*.*

**Fig. S2: *cry1*, *uvr8*, and *cry1 uvr8* mutants in overwintering and non-overwintering cohorts.**

**A**. Images of herbaria specimens with flowers and fruits in summer, collected in the Zurich Area of Switzerland on 14 July 1930 (left) and 5 and 20 July 1932 (right) (Herbarium of the Botanical Garden in Zurich).

**B,C**. Bar chart of survival rate (**B**) and box plots of fruit number (**C**) from five independent experiments in field conditions. Four experiments were performed in Zurich (white text box) and one in the Canton of Grisons (“Mountain Cohort”, light blue text box, indicated at the bottom) with different transfer stages from left to right: Non-overwintering cohort 1, seedling (indicated as pregrown); Overwintering cohort 1, seedling; Overwintering cohort 2, seedling; Overwintering cohort 2, seed; Mountain cohort, seedling. **B**: Survival rate of individuals at the time of bolting; **C**: Fruit number in Non-overwintering cohort 1 and Overwintering cohort 1. In both Overwintering cohorts 2, fruit number was not measured, and in Mountain cohort, measurement of fruit number was not possible due to herbivory (“no data”). Asterisk represents significant difference (p = 0.002). Genotypes and number of replicates by experiment, from left to right as plotted (B and C): Overwintering cohort 1, seedling: L*er*, n = 15; *uvr8-1*, n = 14. Overwintering cohort 2, seedling: L*er*, n = 12; *uvr8-1*, n = 12. Overwintering cohort 2, seed: L*er*, n = 19; *uvr8-1*, n = 19. Non-overwintering cohort 1, seedling: L*er*, n = 17; *uvr8-1*, n = 16. Mountain cohort, seedling: L*er*, n = 8; *uvr8-1*, n = 8.

**D.** Images of field-grown plants from Overwintering cohort 2 after 114 days in the open field (L*er* background). Two wild type (WT) L*er* plants are shown on the left and two *uvr8-1* plants on the right. Scale bar: 1 cm.

**E**. Boxplot of aboveground biomass (fresh weight) (in mg) in Overwintering cohort 3 after 4 months (123 d). Four genotypes of two different backgrounds (left: L*er*, right: Col-0) were included. Genotypes and number of replicates (from left to right, as plotted): WT (L*er*), n = 6; *uvr8* (*uvr8-1*), n = 6; *cry1* (*hy4*-*2.23N*), n = 6; *cry1uvr8* (*hy4-2.23N uvr8-1*), n = 4; WT (Col-0), n = 7; *uvr8* (*uvr8-19*), n = 6; *cry1* (*cry1*-304), n = 6; *cry1uvr8* (*cry1-304 uvr8-19*), n = 4.

**F**. Bar chart of survival rate in Overwintering cohort 3 at time of harvest. Four genotypes of two different backgrounds (left: L*er*, right: Col-0) were included. Genotypes and number of replicates (from left to right, as plotted): WT (L*er*); *uvr8* (*uvr8-1*); *cry1* (*hy4-2.23N*); *cry1uvr8* (*hy4-2.23N uvr8-1*); WT (Col-0); *uvr8* (*uvr8-19*); *cry1* (*cry1*-304); *cry1uvr8* (*cry1-304 uvr8-19*); initial number of replicates: n = 30 for all. Red arrows indicate no surviving plants in *cry1uvr8* genotypes of both backgrounds.

**G.** Relative leaf anthocyanin accumulation in Non-overwintering cohort 3. Leaf material was collected after 60 days in open field conditions, weighed directly after harvest, and then placed into anthocyanin extraction buffer. Four genotypes of Col-0 background were analyzed, with 5 replicates for each. Below plots, and corresponding images of field-grown plants 45 days after field transfer in Non-overwintering cohort 3 before the collection of tissue for anthocyanin accumulation assessment. Four genotypes in the Col-0 background are shown in plots and photographs, from left to right: WT (Col-0), *uvr8* (*uvr8-19*), *cry1* (*cry1-304*), *cry1uvr8* (*cry1-304 uvr8-19*) (five replicates each). Scale bars: 1 cm.

**Fig. S3: Fitness components of *cry1 uvr8* mutants in non-overwintering conditions**

**A**. Boxplot of inflorescence dry weight in Non-overwintering cohort 3 with different UV levels. Seedlings at the five-leaf stage were transferred to the field. Genotypes and number of replicates included per experiment (first number refers to Low-UV, second number to UV-med, third to no UV-attenuation in open, natural solar irradiance) from left to right as plotted: WT (L*er*), n = 4 | 4 | 4; *uvr8* (*uvr8-1*), n = 4 | 4 | 4; *cry1* (*hy4-2.23N*), n = 4 | 4 | 6; *cry1 uvr8* (*hy4-2.23N uvr8-1*), n = 4 | 4 | 4; WT (Col-0), n = 4 | 4 | 11; *uvr8* (*uvr8-19*), n = 4 | 4 | 9; *cry1* (*cry1-304*), n = 4 | 4 | 11; *cry1 uvr8* (*cry1-304 uvr8-19*), n = 4 | 4 | 10. The significance level of the *cry1* x *uvr8* effect (statistical interaction) is given in each individual plot.

**B.** Scatterplot of inflorescence dry weight and fruit number in Non-overwintering cohort 2 by treatment, totaling 297 plants in Low-UV (left window) and 276 in UV-med (right window) of all genotypes and backgrounds in this experiment. Fruits were weighed after drying at 60°C for 24 h and counted afterwards. Results of Pearson’s correlation coefficient (adjusted R^2^) are indicated at the bottom of the corresponding scatterplot. Color legend indicates genotype of both backgrounds (L*er*, Col-0).

**C**. Boxplot of inflorescence dry weight in Non-overwintering cohort 2 with different UV levels. Seeds were transferred to the field. Genotypes and number of replicates included per experiment (first number refers to Low-UV, and second number to UV-med from left to right as plotted: WT (L*er*), n = 32 | 35; *uvr8* (*uvr8-1*), n = 34 | 28; *cry1* (*hy4-2.23N*), n = 40 | 28; *cry1 uvr8* (*hy4-2.23N uvr8-1*), n = 43 | 40; WT (Col-0), n = 36 | 33; *uvr8* (*uvr8-19*), n = 33 | 36; *cry1* (*cry1-304*), n = 40 | 35; *cry1 uvr8* (*cry1-304 uvr8-19*), n = 39 | 41. The significance level of the *cry1* x *uvr8* effect (statistical interaction) is given in blue at the top of the plot.

**Fig. S4: Double mutants with and without supplemental UV-B irradiation**

**A,B**. Photographs of 28-day-old wild type (Col-0), *uvr8-19* (*uvr8*), *cry1-304* (*cry1*), and *cry1-304* *uvr8-19* (*cry1* *uvr8*) plants grown **(A)** with supplemental UV-B (+UV-B”, ca 40% of natural UV-B in July 2018), **(B)** without UV-B (UV-B-exclusion) in Chamber cohort 7. Scale bars = 5 cm.

**C**. Bar chart of survival rate after 35 days under +UV-B and UV-B-exclusion conditions (Chamber cohort 7). Six replicates of all Col-0 background genotypes were included, from left to right as plotted: WT (Col-0); *uvr8* (*uvr8-19*); *cry1* (*cry1-304)*; *cry1 uvr8* (*cry1-304 uvr8-19*).

**D**. Survival rate after 35 days under Chamber-UV and UV-B-exclusion conditions (Chamber cohort 6). Twelve replicates per genotype in Col-0 and L*er* backgrounds were grown. Genotypes from left to right as plotted: wild type (WT; L*er*); *uvr8* (*uvr8-1*); *cry1* (*hy4-2.23N*); *cry1 uvr8* (*hy4-2.23N uvr8-1*); WT (Col-0); *uvr8* (*uvr8-19*); *cry1* (*cry1-304)*; *cry1 uvr8* (*cry1-304 uvr8-19*).

**E**. Image of UV-B-exclusion chamber setup. One shelf is covered in a box-like frame with UV filter film panels (Rosco #226). The red arrow indicates the front panel where the UV-blocking filter was cut into 25-cm strips to allow airflow and manual watering. The top and shorter side panels were fixed and the back panel was open (wall ventilation). The measured UV levels were below the threshold of the sensor for UV-B (UV-microlog).
