# Supplementary material for: UV RESISTANCE LOCUS 8–Mediated UV-B Response Is Required Alongside CRYPTOCHROME 1 for Plant Survival in Sunlight under Field Conditions
Source: Plant Cell Physiol. 2023 Sep 27;65(1):35–48. doi: 10.1093/pcp/pcad113 (PMC10799719; doi:10.1093/pcp/pcad113)
Supplement: pcad113_Supp [file pcad113_supp.zip › suppl_data.zip!/suppl_data/pcp-2023-e-00210-File007.docx]

| **NASC ID** | **Affected gene** | **Mutation** | **Background** | **Genotype** | **Mutagen** | **Published** |
| --- | --- | --- | --- | --- | --- | --- |
| N6673 | *-* | - | Col | Col-0 | - | - |
| - | *UVR8* | Trp-144 to Stop | Col | *uvr8-19* | EMS | Podolec *et al*., 2021b |
| - | *CRY1* | 481-bp deletion (5′ region) | Col | *cry1-304* | fast neutrons | Mockler *et al*., 1999 |
| - | *CRY1, UVR8* | see *cry1-304* and *uvr8-19* | Col | *cry1-304 uvr8-19* | see *cry1-304* and *uvr8-19* | this study |
| N20 | *-* | - | L*er* | L*er* | - | - |
| - | *UVR8* | 15-bp deletion | L*er* | *uvr8-1* | fast neutrons | Kliebenstein *et al*., 2002 |
| N70 | *CRY1* | ca. 300-bp deletion | L*er* | *hy4-2.23N* | fast neutrons | Ahmad & Cashmore, 1993 |
| - | *CRY1, UVR8* | see *hy4-2.23N* and *uvr8-1* | L*er* | *hy4-2.23N uvr8-1* | see *hy4-2.23N* and *uvr8-1* | Tissot and Ulm, 2020 |
| N28823 | *-* | - | Ws | Ws | - | - |
| - | *UVR8* | Gln-124 to Stop | Ws | *uvr8-7* | EMS | Favory *et al*., 2009 |

**Table S1: Information of plant genotypes and accessions used in this study.** NASC ID refers to available seeds at the Nottingham Arabidopsis Stock Center (http://arabidopsis.info). Affected gene indicates the target gene, Mutation column indicates the type and position of mutations on the target gene. Background refers to the original wild type. Genotype shows the name of the genotype used in this manuscript. Mutagen describes the method used to create the mutants. EMS = ethyl methanesulfonate. A “-“ in the first column indicates no entry in the NASC database, or otherwise that this genotype is a wild type plant (no mutation).

| **Experiment** | **Duration (start–end)** | **Initial number of replicates** | **Transfer as** | **UV-B level** | **Disrupted experiment (no data)** | **Figures** |
| --- | --- | --- | --- | --- | --- | --- |
| **Non-overwintering cohort 1** | 05–08.2014 | 18 | seedling | natural (full) | - | Fig. S2 B-C |
| **Non-overwintering cohort 2** | 05.2018 | 48 | seed | natural (full) | yes (heavy rain) | - |
| **Non-overwintering cohort 2** | 05–07.2018 | 48 | seed | UV-med (Rosco #130) | - | Fig. 3; Fig. S3B-C; Fig. 4 |
| **Non-overwintering cohort 2** | 05–07.2018 | 48 | seed | Low-UV (Rosco #226) | - | Fig. 3; Fig. S3B-C; Fig. 4 |
| **Non-overwintering cohort 3** | 08–11.2018 | 18 | seedling | natural (full) = open | - | Fig. S2G, Fig. S3A |
| **Non-overwintering cohort 3** | 08–11.2018 | 8 | seedling | UV-med (Rosco #130) | - | Fig. S3A |
| **Non-overwintering cohort 3** | 08–11.2018 | 8 | seedling | Low-UV (Rosco #226) | - | Fig. S3A |
| **Overwintering cohort 1** | 10.2014–04.2015 | 15 | seedling | natural (full) | - | Fig. S2B-C |
| **Overwintering cohort 2** | 10.2015 – 04.2016 | 20 | seed | natural (full) | - | Fig. S2B,D |
| **Overwintering cohort 2** | 10.2015 – 04.2016 | 12 | seedling | natural (full) | - | Fig. S2B |
| **Overwintering cohort 3** | 10.2018–04.2019 | 30 | seed | natural (full) | - | Fig. 2, Fig. S2E-F |
| **Mountain cohort 1** | 06–08.2014 | 8 | seedling | natural (full) | - | Fig. S2B |
| **Mountain cohort 2** | 06.2015 | 20 | seedling | natural (full) | yes | - |
| **Mountain cohort 3** | 09.2014 | 20 | seedling | natural (full) | yes | - |
| **Chamber cohort 1** | 02–03.2014 | 28 | - | WL fluorescent tubes | - | Fig. 1 |
| **Chamber cohort 2** | 07–08.2014 | 8 | - | WL fluorescent tubes | - | Fig. S1A-C |
| **Chamber cohort 3** | 02–04.2016 | 8 | - | WL fluorescent tubes | - | Fig. S1A |
| **Chamber cohort 4** | 02–03.2019 | 10 | - | WL fluorescent tubes | - | Fig. S1D |
| **Chamber cohort 5** | 05–07.2018 | 14 | - | Rosco #226  (UV-B exclusion) | - | Fig. S1E |
| **Chamber cohort 6** | 02–04.2019 | 12 | - | WL fluorescent tubes | - | Fig. S4D |
| **Chamber cohort 6** | 02–04.2019 | 12 | - | Rosco #226  (UV-B exclusion) | - | Fig. S4D-E |
| **Chamber cohort 7** | 02–04.2019 | 6 | - | WL fluorescent tubes | - | Fig. S4B,C |
| **Chamber cohort 7** | 02–04.2019 | 6 | - | supplemental UV-B (+UV-B) | - | Fig. S4A,C |

**Table S2: Details of experiments in this study.**  Each row refers to one experiment. The “Duration (start-end)” column indicates the month the experiment started and the month it was concluded. The “Initial number of replicates” column refers to the number of pots per genotype that were transferred to the field or grown in the growth chambers. The “Transfer as” column displays the growth stage for the individual plants when transferred to the field; “-“ if plants stayed in the growth chamber. In the “UV-B level” column, the different UV-B levels are indicated (note WL fluorescent tubes mean low levels of UV-B intrinsic to white light fluorescent tubes). The ”Disrupted experiment (no data)” column shows if an experiment did (“-“) or did not harbor any data (“yes”). In the “Figures” column, figure panels showing results from the corresponding experiment are given.

| **Trait** | **Subset** | **Figure** | **Experiment** | **Model type** | **ANOVA test** | **Explanatory variables** | **Random Factors** | **Statistics value** | **p-value** |
| --- | --- | --- | --- | --- | --- | --- | --- | --- | --- |
| Fruit number | - | Fig. 1 | Chamber cohort 1 | GLMM, quasi-poisson | Type II | uvr8_mutant | **background**, block | 80.66 | **< 2.20e-16** |
| Fruit number | L*er* background | Fig.1 | Chamber cohort 1 | LM | Type II | uvr8_mutant | - | 30.48 | **1.21e-06** |
| Fruit number | Ws background | Fig.1 | Chamber cohort 1 | LM | Type II | uvr8_mutant | - | 49.29 | **4.18e-09** |
| Fruit number | - | Fig. S1A | Chamber cohort 2,3 | GLMM, quasi-poisson | Type II | uvr8_mutant | **background**, block | 23.24 | **1.43e-06** |
| Fruit number | L*er* background | Fig. S1A | Chamber cohort 2 | LM | Type II | uvr8_mutant | - | 7.31 | **0.018** |
| Fruit number | L*er* background | Fig. S1A | Chamber cohort 3 | LM | Type II | uvr8_mutant | - | 5.70 | **0.017** |
| Fruit number | Ws background | Fig. S1A | Chamber cohort 2 | GLM, quasi-poisson | Type II | uvr8_mutant | - | 9.85 | **0.002** |
| Fruit length | - | Fig. S1B | Chamber cohort 2 | LMM | Type II | uvr8_mutant | background, block | 0.39 | 0.532 |
| Seeds per fruit | - | Fig. S1C | Chamber cohort 2 | LMM | Type II | uvr8_mutant | block | 0.31 | 0.578 |
| Aboveground biomass (fresh weight) | - | Fig. S1D | Chamber cohort 4 | GLMM, quasi-poisson | Type II | uvr8_mutant | background, block | 6.00 | **0.0143** |
| Aboveground biomass (fresh weight) | L*er* background | Fig. S1D | Chamber cohort 4 | LM | Type II | uvr8_mutant | - | 3.87 | **0.049** |
| Aboveground biomass (fresh weight) | Col background | Fig. S1D | Chamber cohort 4 | LM | Type II | uvr8_mutant | - | 6.08 | **0.014** |
| Fruit number | - | Fig. S1E | Chamber cohort 5 | LMM | Type II | uvr8_mutant | block | 1.64 | 0.201 |
| Seedling establishment | - | Fig. 2A | Overwintering cohort 3 | GLMM, binomial | Type III | cry1_mutant x uvr8_mutant | background, block | 10.75 | **0.001** |
| Fruit number | - | Fig. 2C | Overwintering cohort 3 | GLMM, quasi-poisson | Type III | cry1_mutant x uvr8_mutant | background, block | 303.10 | **< 2.20e-16** |
| Survival (rate) (Zurich) | - | Fig. S2B | Non-overwintering cohort 1 (seedling stage) | GLM, binomial | Type II | uvr8_mutant | - | 2.91 | 0.088 |
| Survival (rate) (Zurich) | - | Fig. S2B | Overwintering cohort 1 (seedling stage) | GLM, binomial | Type II | uvr8_mutant | - | 0.00 | 1 |
| Survival (rate) (Zurich) | - | Fig. S2B | Overwintering cohort 2 (seedling stage) | GLM, binomial | Type II | uvr8_mutant | - | 0.20 | 0.653 |
| Survival (rate) (Zurich) | - | Fig. S2B | Overwintering cohort 2 (seed stage) | GLM, binomial | Type II | uvr8_mutant | - | 2.88 | 0.089 |
| Survival (rate) (Mountain cohort) | - | Fig. S2B | Mountain cohort 1(seedling stage) | GLM, binomial | Type II | uvr8_mutant | - | 9.29 | **0.002** |
| Fruit number | - | Fig. S2C | Non-overwintering cohort 1 (seedling stage) | LM | Type II | uvr8_mutant | - | 0.03 | 0.963 |
| Fruit number | - | Fig. S2C | Overwintering cohort 1 (5-leaf stage) | GLM | Type II | uvr8_mutant | - | 0.00 | 0.945 |
| Aboveground biomass (fresh weight) | - | Fig. S2E | Overwintering cohort 3 | GLMM, quasi-poisson | Type III | cry1_mutant x uvr8_mutant | background, block | 16.25 | **5.54e-05** |
| Survival (rate) | - | Fig. S2F | Overwintering cohort 3 | GLMM, binomial | - | - | - | (complete separation due to synthetic lethality ) |  |
| Anthocyanin accumulation | - | Fig. S2G | Non-overwintering cohort 3 | GLMM, Gamma | Type III | cry1_mutant x uvr8_mutant | background, block | 30.99 | **2.59e-08** |
| Fruit number | - | Fig. 3C | Non-overwintering cohort 2 | GLMM, quasi-poisson | Type III | UV-B condition x cry1_mutant x uvr8_mutant | background, block | 4.35 | **0.037** |
| Fruit number | UV-med | Fig. 3C | Non-overwintering cohort 2 | GLMM, quasi-poisson | Type III | cry1_mutant x uvr8_mutant | background, block | 17.26 | **3.26e-05** |
| Fruit number | Low-UV | Fig. 3C | Non-overwintering cohort 2 | GLMM, negative binomial | Type III | UV-B condition x cry1_mutant x uvr8_mutant | background, block | 0.30 | 0.582 |
| Inflorescence dry weight | Low-UV | Fig. S3A | Non-overwintering cohort 3 (seedling stage) | GLMM, negative binomial | Type III | cry1_mutant x uvr8_mutant | **background** | 3.20 | 0.074 |
| Inflorescence dry weight | UV-med | Fig. S3A | Non-overwintering cohort 3 (seedling stage) | LMM | Type III | cry1_mutant x uvr8_mutant | background | 9.60 | **0.002** |
| Inflorescence dry weight | open | Fig. S3A | Non-overwintering cohort 3 (seedling stage) | GLMM, quasi-poisson | Type III | cry1_mutant x uvr8_mutant | background, block | 18.94 | **1.35e-05** |
| Inflorescence dry weight | - | Fig. S3C | Non-overwintering cohort 2 | GLMM, quasi-poisson | Type III | UV-B condition x cry1_mutant x uvr8_mutant | **background, block** | 4.33 | **0.038** |
| Inflorescence dry weight | Low-UV | Fig. S3C | Non-overwintering cohort 2 | GLMM, negative binomial | Type III | cry1_mutant x uvr8_mutant | background, block | 0.07 | 0.790 |
| Inflorescence dry weight | UV-med | Fig. S3C | Non-overwintering cohort 2 | GLMM, quasi-poisson | Type III | cry1_mutant x uvr8_mutant | background, block | 13.25 | **2.72e-04** |
| Survival (rate) | + UV-B | Fig. S4C | Chamber cohort 7 | GLMM, binomial | Type III | cry1_mutant x uvr8_mutant | block | 114.09 | **< 2.20e-16** |
| Survival (rate) | UV-B-exclusion | Fig. S4C | Chamber cohort 7 | - | - | - | - | constant response (all survived) |  |

**Table S3: Statistical analysis.** Models with *uvr8*_mutant and/or *cry1*_mutant as explanatory variables (fixed factor) are shown, testing the effect of mutants on single fitness components as given in the first column. The second column indicates subsets of data for analysis. Figures in which the trait is visualized are indicated in the third column. Information on the experiment can be found in the fourth column. Columns five to eight bear information on the selected model. Interactions are shown as “x” between factors. The second to right and rightmost column show Statistics (F-statistics for LM, Chi-squared statistics for other model types) and p-value from the analysis, respectively. “-” indicates an absence in the respective column. Significant effects (p < 0.05) of random factors and significant p-values are given in bold letters.

| **Treatment** | **Daily UV-B dose*** | **UV-B compared to "July UV ZH"** | **Date** | **Experiment** |
| --- | --- | --- | --- | --- |
| July UV ZH | 59.07 | 100% | 07-09-2018 | Non-overwintering cohort 2 |
| September UV ZH | 34.69 | 58.7% | 09-05-2018 | Non-overwintering cohort 3 |
| February UV ZH | 13.92 | 23.6% | 02-17-2019 | Overwintering cohort 3 |
| July UV Mountain cohort | 88.21 | 149.3% | 07-05-2014 | Mountain cohort |
| UV-med (filter) | 15.31 | 25.9% | 07-09-2018 | Non-overwintering cohort 2 "UV-med" |
| Low-UV (filter) | 0.40 | 0.7% | 07-09-2018 | Non-overwintering cohort 2 "Low-UV" |
| Chamber UV | 0.86** | 1.5% | 11-11-2018 | Chamber cohort 1-4,6,7 |
| UV-B exclusion (filter) | undetectable | - | 11-27-2018 | Chamber cohort 5-6 |
| +UV-B | 23.85 | 40.4% | 12-02-2019 | Chamber cohort 7, "+UV-B" |

^*^ erythemally weighted UV-B, mW/m^2^/day; measure with UVMicrolog (sglux)

^**^ integral of light period values under LD conditions (16-h light)

**Table S4: UV-B conditions in different treatments.** Integral values of UV-B measurements for one sunny day (00:00-00:00 in 10-minute intervals) in field condition per experiment are shown (July UV ZH, September UV ZH, February UV ZH, July UV Mountain cohort). In addition, integral values from chamber environments are given for a similar interval. Integrals were always compared to July UV ZH (value = 59.07). UV-med indicates filtering with Rosco #130 film whereas Low-UV (in the field) and UV-B-exclusion (in the chambers) indicate filtering with Rosco #226 film. +UV-B indicates supplemental UV-B provided with Philips “TL20W/01RS” narrowband UV-B tubes. Experiment column: experiment name in Table S2, indicating in which experiments UV-B treatments were used. ZH: Zurich.

**Table S5^*^: Count_QC-raw-count.** The first column in the first sheet indicates gene numbers of *Arabidopsis thaliana*. Other columns are read count of each sample. Sample names are explained in the second sheet.

*This table is a separate worksheet

**Table S6^*^: Gene expression statistics.** baseMean: average of the normalized count values, divided by size factors (over all samples), log2Foldchange: is estimate of the effect size, lfcSE: standard error of the log2Foldchange (uncertainty estimate), stat: Wald statistic value, pvalue: Wald test p-value, padj: Benjamini-Hockberg (BH) adjusted p-values.

*This table is a separate worksheet

**Table S7^*^: Differentially expressed genes (DEG) list – UV-med.** DEG of mutants compared to wild types in UV-med condition of Non-overwintering cohort 2. All genes with false-discovery-rate (FDR) ≤ 0.05 are retained and shown with FDR and log2 ratio of expression. Positive log2 ratio values indicate higher expression than wild type, whereas negative log2 ratio values indicate lower expression.

*This table is a separate worksheet

**Table S8^*^: Gene Ontology (biological process) analysis – UV-med.** Gene Ontology analysis of the data shown in Table S7. Categories obtained with topGO v2.48.0 with the elim algorithm and Fisher statistics. Categories were filtered for gene number (between 10 and 999) and elim <=0.05.

*This table is a separate worksheet

**Table S9^*^: Genes showing significant statistical interaction of the mutations *UVR8* and *CRY1* in RNA expression – UV-med.** The data in UV-med condition of Non-overwintering cohort 2 were analyzed. All genes with padj ≤ 0.05 are shown. baseMean: average of the normalized count values, divided by size factors (over all samples), log2Foldchange: is estimate of the effect size, lfcSE: standard error of the log2Foldchange (uncertainty estimate), stat: Wald statistic value, pvalue: Wald test p-value, padj: Benjamini-Hockberg (BH) adjusted p-values.

*This table is a separate worksheet

**Table S10*: Genes showing significant statistical interaction of the mutations *UVR8* and *CRY1* in RNA expression – Low-UV.** The data in low UV condition of Non-overwintering cohort 2 were analyzed. All genes with padj ≤ 0.05 are shown. baseMean: average of the normalized count values, divided by size factors (over all samples), log2Foldchange: is estimate of the effect size, lfcSE: standard error of the log2Foldchange (uncertainty estimate), stat: Wald statistic value, pvalue: Wald test p-value, padj: Benjamini-Hockberg (BH) adjusted p-values.

*This table is a separate worksheet

**Table S11*: Genes showing significant statistical interaction of the mutations *UVR8* and *CRY1* – UV-B-exclusion.** The data in UV-B-excolusion were analyzed. All genes with padj ≤ 0.05 are shown. All genes with padj ≤ 0.05 are shown. baseMean: average of the normalized count values, divided by size factors (over all samples), log2Foldchange: is estimate of the effect size, lfcSE: standard error of the log2Foldchange (uncertainty estimate), stat: Wald statistic value, pvalue: Wald test p-value, padj: Benjamini-Hockberg (BH) adjusted p-values.

*This table is a separate worksheet

| **Gene ID** | **Gene name** | **UV-med** | **Low-UV** |
| --- | --- | --- | --- |
| AT1G06570 | *HPD* | **4.34 ***** | 1.84 |
| AT4G32770 | *VTE1* | **3.29 *** | 1.02 |
| AT5G53970 | *TAT1* | **2.92 *** | 0.57 |
| AT2G29570 | *PCNA2* | **3.58 **** | 1.85 |
| AT4G02070 | *MSH6* | **3.31 *** | 1.83 |
| AT3G18524 | *MSH2* | **3.08 *** | 1.80 |

**Table S12**: **Statistics for differentially expressed genes (DEG) in Figure 4C**. The Gene ID and Gene name columns are based on TAIR annotation. The values in column UV-med and Low-UV refer to the statistical interaction in the two respective conditions and show the absolute z-score values for the statistical interaction term from the test implemented in DESeq2. Bold numbers indicate statistical significance, asterisks refer to significance levels (* = p ≤ 0.05, ** = p ≤ 0.01, *** = p ≤ 0.001).

**Table S13*: Analysis of Gene Ontology (GO) of biological process for reduced-expression genes with significant interaction of the mutations *UVR8* and *CRY1* in the UV-med condition. The result of** GO enrichment analysis with 513 genes with log2 values equal to or lower than -1 in Table S10 is shown. First and second column entries are GO ID and the first 40 letters of the GO term, respectively. "Annotated” refers to number of annotated genes in the TAIR10 genome release that are associated with the GO-term, "Significant" refers to the number of genes found in the gene list from our analysis. "Expected " is the number of genes expected in the gene list based on the total number of genes in the TAIR10 genome. The list is composed of GO categories with more than 10 and less than 1’000 annotated genes. The given "elim” value corresponds the p-value based on the conservative elim fisher algorithm in TopGO.

*This table is a separate worksheet

**Table S14*: Analysis of Gene Ontology (GO) of biological process for increased-expression genes with significant interaction of the mutations *UVR8* and *CRY1* in the UV-med condition. The result of** GO enrichment analysis with 513 genes with log2 values equal to or higher than 1 in Table S10 is shown. First and second column entries are GO ID and the first 40 letters of the GO term, respectively. "Annotated" refers to number of annotated genes in the TAIR10 genome release that are associated with the GO-term, "Significant" refers to the number of genes found in the gene list from our analysis. "Expected" is the number of genes expected in the gene list based on the total number of genes in the TAIR10 genome. The list is composed of GO categories with more than 10 and less than 1’000 annotated genes. The given "elim” value corresponds to the p-value based on the conservative elim fisher algorithm in TopGO.

*This table is a separate worksheet

| **Genotype** | ***uvr8*_mutant** | ***cry1*_mutant** |
| --- | --- | --- |
| *uvr8-1* | yes | no |
| *uvr8-7* | yes | no |
| *uvr8-19* | yes | no |
| *hy4-2.23N* | no | yes |
| *cry1-304* | no | yes |
| *hy4-2.23N uvr8-1* | yes | yes |
| *cry1-304 uvr8-19* | yes | yes |
|  |  |  |
| L*er* | no | no |
| Col-0 | no | no |
| Ws | no | no |

**Table S15: Scoring of genotypes as used in the model for statistical analysis.** Two different factors, *uvr8_mutant* and *cry1_mutant*, were created to test the effect of each mutant separately as well as to study interaction effects between mutations. These factors were included in the statistical analysis model in every experiment, and interactions were included when double mutant genotypes were present in the experiment.
